# Hemolymph of triatomines presents fungistatic activity against *Cryptococcus neoformans* and improves macrophage function through MCP-I/TNF-α increase

**DOI:** 10.1590/1678-9199-JVATITD-2021-0124

**Published:** 2022-07-18

**Authors:** Luísa Menezes-Silva, Jonatas da Silva Catarino, Laura Caroline de Faria, Bárbara Cristina Pizzolante, Leonardo Eurípedes Andrade-Silva, Marcos Vinicius da Silva, Virmondes Rodrigues, Helioswilton Sales-Campos, Carlo José Freire Oliveira

**Affiliations:** 1 Laboratory of Immunology and Bioinformatics, Department of Microbiology, Immunology and Parasitology, Institute of Biological and Natural Sciences, Federal University of Triângulo Mineiro, Uberaba, MG, Brazil.; 2 Department of Immunology, Institute of Biomedical Sciences, University of São Paulo (USP), São Paulo, SP, Brazil.; 3 Department of Comparative Medicine, Yale University School of Medicine, New Haven, CT, United States.; 4 Department of Biosciences and Technology, Institute of Tropical Pathology and Public Health, Federal University of Goiás, Goiânia, GO, Brazil.

**Keywords:** Hemolymph, Triatomines, Macrophages, Meccus pallidipennis, Rhodnius prolixus, Cryptococcus neoformans

## Abstract

**Background:**

Triatomines are blood-feeding arthropods belonging to the subfamily Triatominae (Hemiptera; Reduviidae), capable of producing immunomodulatory and water-soluble molecules in their hemolymph, such as antimicrobial peptides (AMPs). In this work, we evaluated the antifungal and immunomodulatory activity of the hemolymph of *Meccus pallidipennis* (MPH) and *Rhodnius prolixus* (RPH) against *Cryptococcus neoformans*.

**Methods:**

We assessed the activity of the hemolymph of both insects on fungal growth by a minimum inhibitory concentration (MIC) assay. Further, RAW 264.7 macrophages were cultivated with hemolymph and challenged with *C. neoformans*. Then, their phagocytic and killing activities were assessed. The cytokines MCP-1, IFN-γ, TNF-α, IL-10, IL-12, and IL-6 were measured in culture supernatants 4- and 48-hours post-infection.

**Results:**

Both hemolymph samples directly affected the growth rate of the fungus in a dose-dependent manner. Either MPH or RPH was capable of inhibiting fungal growth by at least 70%, using the lowest dilution (1:20). Treatment of RAW 264.7 macrophages with hemolymph of both insects was capable of increasing the production of MCP-I and TNF-α. In addition, when these cells were stimulated with hemolymph in the presence of *C. neoformans*, a 2- and a 4-fold increase in phagocytic rate was observed with MPH and RPH, respectively, when compared to untreated cells. For the macrophage killing activity, MPH decreased in approximately 30% the number of viable yeasts inside the cells compared to untreated control; however, treatment with RPH could not reduce the total number of viable yeasts. MPH was also capable of increasing MHC-II expression on macrophages. Regarding the cytokine production, MCP-I and TNF-α, were increased in the supernatant of macrophages treated with both hemolymphs, 4 and 48 hours after stimulation.

**Conclusion:**

These results suggested that hemolymph of triatomines may represent a source of molecules capable of presenting antifungal and immunomodulatory activity in macrophages during fungal infection.

## BACKGROUND

Insects, from the phylum Arthropoda, constitute the largest number of species in the animal kingdom [1]. They are exposed to different microorganisms in distinct habitats due to their worldwide distribution. Some microorganisms develop symbiotic relationships with arthropods, without causing any harm; instead, others can be pathogenic [2,3]. For this reason, these invertebrates had to develop several mechanisms of defense throughout their evolution [4]. The first line of defense of insects is formed by the chitosan exoskeleton and cuticle [5]. When a microorganism overcomes these barriers, other effector mechanisms are used to fight infection. The insects have only innate immunity, which is composed of cellular and humoral immune responses [6-8]. Hemocytes and cells in the fatty body are the major cellular components. The humoral response is represented by antimicrobial peptides (AMPs), molecules comparable to the complement system in humans, besides enzymatic and coagulation cascades [6,8].

Triatomines belong to the subfamily Triatominae (Hemiptera; Reduviidae), popularly known as “kissing bugs”, and have evidenced medical importance by their role as vectors of *Trypanosoma cruzi*, the causative agent of Chagas disease [9]. So far, approximately 150 species have been identified, from five tribes, and two fossil species [10-13]. Among them, it is possible to highlight two species: *Meccus pallidipennis* and *Rhodnius prolixus*. The *M. pallidipennis* is a key vector of *T. cruzi* mainly in North and Central Americas [14-16]. On the other hand, *R. prolixus* is one of the most important vectors in the South and Central Americas [17,18]. Because of their wide geographical distribution, triatomines are constantly exposed to distinct environments and microorganisms throughout their lifespan. Several AMPs from triatomines have been isolated and characterized, and their mechanism of action seems to be dependent on the pathogen (bacteria, viruses, fungi, and protozoa) [2]. In the environment or during blood feeding, these molecules must be able to neutralize and kill harmful microorganisms. 

Among the different classes of microorganisms, fungi have received attention due to their drug resistance mechanisms, their similarities with eukaryotic cells, the production of mimicking host molecules, and the presence of a cell wall [19]. *Cryptococcus neoformans* is a cosmopolitan microorganism with global distribution, found in contaminated soil, decomposing wood, or bird fecal matter [20]. The inhalation of basidiospores or dehydrated yeasts of *C. neoformans* by immunocompromised individuals may result in a systemic infection, called cryptococcosis. After entering the lungs, the yeasts can enter the hematologic route and have a tropism for the central nervous system, thus representing a threat [21,22]. Once at the respiratory tract, the alveolar phagocytic cells are the first line of defense against *C. neoformans* [20,23]. In this regard, alveolar macrophages are one of the key cells against *Cryptococcus* spp. infections [24-27].

Recently, therapies with a concomitant activity towards microorganisms and the immune system have been surveyed in drug discovery research [28]. Thus, therapeutic approaches aiming at activating the immune system rather than just killing the pathogen may represent more promising and effective candidates. A previous work from our group has demonstrated that the saliva and the hemolymph of hematophagous insects have components that differentially modulate the immune system of mammalians [29,30]. The purpose of this work, therefore, is to evaluate the relationship between molecules in the hemolymph of triatomines, *M. pallidipennis* and *R. prolixus*, with antifungal and immunomodulatory activities during *C. neoformans* infection.

## METHODS

### Triatomines and hemolymph collection

Triatomines were obtained from the insectary of the Federal University of Triângulo Mineiro. Before collection, triatomines were cleaned with ethanol 70%, and saline. The pairs of legs were sectioned, and the hemolymph that overflowed was removed with a pipette. For the conservation of hemocytes and hemolymph, the extract was collected in the presence of sodium citrate buffer/pH 4.6, 1:1. Then, the hemolymph was stored at -80°C until use. The protein dosage was performed prior to use in a NanoDrop™ 2000/2000c (Thermo Fisher Scientific, Waltham, Massachusetts, USA), at 280 nm, to avoid sample loss. At the time of use, the total volume of hemolymph was diluted in RPMI medium (RPMI 1640, Sigma-Aldrich, San Luis, Missouri, USA), and the content was filtered at 22μm to exclude contamination. From this moment on, the hemolymph is described as follows: *Meccus pallidipennis* hemolymph (MPH) and *Rhodnius prolixus* hemolymph (RPH).

### Growth inhibition assay

The fungicidal activity of hemolymph was accessed by the analysis of the fungi grow rate in RPMI 1640 medium supplemented with L-glutamine, without sodium bicarbonate (Sigma-Aldrich, San Luis, Missouri, USA) and buffered to a pH 7.0 with 0.165 M of MOPS (4-morpholinepropanesulfonic acid, Sigma-Aldrich). The assay was performed in the presence or absence of the hemolymphs, in five different dilutions: 1:20, 1:40, 1:80, 1:160, and 1:320. The assay was performed in sterile 96 well plates, with a final volume of 200 μL, using 1x10^5^ yeasts/well. *C. neoformans* var. *grubii* strain H99 (ATCC® 208821™, Manassas, Virginia, USA) was used for all the experiments. As a positive fungicidal control, Amphotericin B (Sigma-Aldrich, San Luis, Missouri, USA) at 2 μg/mL was used. The fungal growth was accessed after 72 hours, at 37°C, by optical density. The optical density of each well was compared to the positive and negative controls, by giving a scale from 0 to 5, with being 0 attributed to the absence of fungi growth and 5, to 100% of fungi growth.

### Cell culture

For the phagocytic and microbicidal essays, RAW 264.7 (ATCC® TIB-71™) macrophages were used. The macrophages were cultured in complete RPMI medium (RPMI 1640, Sigma-Aldrich, San Luis, Missouri, USA) supplemented with 10% fetal bovine serum - SFB (Gibco™, Gaithersburg, Maryland, USA) and Penicillin/Streptomycin (Gibco™, Gaithersburg, Maryland, USA), at 37°C and 5% CO_2_. About 2 x 10^5^ cells were plated per well and pre-incubated with the hemolymph samples for 4 hours before the beginning of *C. neoformans* infection.

### Hemolymph cell toxicity tests

RAW 264.7 cells were incubated with the hemolymph at the following dilutions: 1:20, 1:40, 1:80, 1:160, and 1:320. Cell toxicity was accessed 4, 8, and 24 hours after the onset of the experiment. After incubation, the plate was read in an inverted microscope (Olympus Corporation, Tokyo, Japan). Cell viability was assessed by counting the number of killed cells in 10 different microscopic fields on a 400-x objective. To further assess the toxicity of hemolymphs, we performed the Annexin-V/Propidium Iodide assay (Annexin V Apoptosis Detection Kit - BD Pharmingen™, New Jersey, USA) after 4 hours of incubation. The cell acquisition was performed in a BD FACSCalibur™ (BD™ Biosciences, Franklin Lakes, New Jersey, USA).

### Phagocytic and fungicidal activity

For the phagocytic and fungicidal activity assessment, a MOI of 10:1 (1 x 10^6^ yeasts) was standardized. For all the assays, the hemolymph was used at 1:80 dilution, according to the data obtained from the toxicity assay. The macrophages were incubated in the presence or absence of hemolymph for 4 hours. Then, the yeasts were placed in culture. Exclusively for phagocytosis experiments, yeasts were heat killed in a water bath for 60’/56° C, as previously described [31,32]. Then, the yeasts were stained with fluorescein 5(6)-isothiocyanate (FITC) (Sigma-Aldrich, San Luis, Missouri, USA) and incubated with the macrophages. 

To determine the killing rate, macrophages were initially incubated with hemolymph for 4 hours. Then, viable live yeasts were added and incubated for 48 hours, at 37ºC and 5% CO_2_. The supernatant was collected and stored at -20°C until use. Each well was washed twice with saline to remove extracellular fungal cells, and the adherent cells were lysed in 500 μL of ultrapure water to release the internalized fungal particles. The macrophage microbicidal activity was analyzed by counting the colony-forming unit (CFU) of the lysate from macrophages in Sabouraud agar (HiMedia, Mumbai, Maharashtra, India). Colony counting was performed using the ImageJ software (National Institute of Health, Bethesda, Maryland, USA‎).

### Flow cytometry analysis

After removing the culture supernatant, 200 μL of PBS buffer + 5% SFB serum was added in each well to block nonspecific staining, for 30’/4°C. Then, each well was washed and incubated with the antibody mix for 30’/4°C. RAW 264.7 macrophages were labeled with antibodies for detection of MHC-II (I-A[b] - Clone AF6-120.1 - BD Biosciences) and CD86 (Clone GL1 - BD Biosciences). Finally, the cells were washed in a PBS buffer and 200 μL of paraformaldehyde 2% was added to fixation. After staining, cells were analyzed in a FACSCalibur™ (BD™ Biosciences, Franklin Lakes, New Jersey, USA). The data were analyzed with the FlowJo™ v10 software (BD™ Biosciences). Gate strategies for the MHC-II/CD86 expression (Additional file 1) and the phagocytosis assay (Additional file 2) are shown in the Supplementary Material section.

### Cytokine production

The production of the following cytokines and chemokines was accessed in culture supernatants by Cytometric Bead Array BD™ Inflammation Kit following the manufacturer’s instruction (BD™ Biosciences): Interleukin-6 (IL-6), Monocyte Chemoattractant Protein-1 (MCP-1), Interferon-γ (IFN-γ), Tumor Necrosis Factor-α (TNF-α), Interleukin-10 (IL-10), and Interleukin-12 (IL-12p70). The data were analyzed with the FCAP Array ™ v3.0 software (BD™ Biosciences).

### Data analysis

Statistical analyzes were performed with GraphPad Prism 7 software (GraphPad Software, CA, USA). First, the groups were analyzed to assess normality. For parametric values, the Student T-test was used for unpaired groups. For non-parametric data, the Mann-Whitney U test was used. For analyzes of more than two groups, the One-Way ANOVA analysis was used for parametric values. Values with *p* <0.05 were considered significant: ^*^ *p* < 0.05; ^**^ *p* < 0.01; ^***^ *p* < 0.001. All the tests were performed in quintuplicate.

## RESULTS

### Triatominae hemolymph presents fungistatic activity against *C. neoformans*

**Figure 1.**
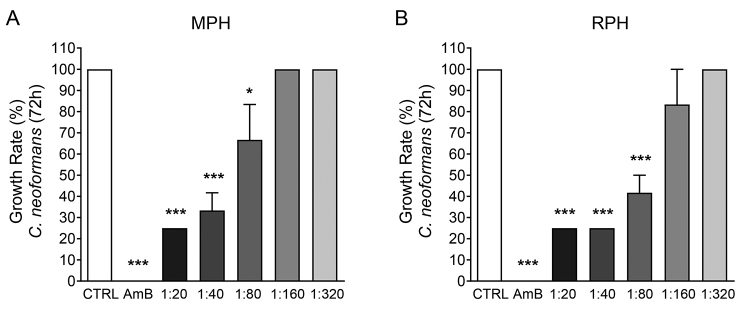
The hemolymph of two hematophagous insects, *Meccus pallidipennis* and *Rhodnius prolixus*, affects in a dose-dependent manner the growth of *Cryptococcus neoformans*. The bars represent the growth rate of *C. neoformans* in the presence of **(A)** *M. pallidipennis* hemolymph (MPH) or **(B)** *R. prolixus* hemolymph (RPH). The fungi were incubated with hemolymph for 72 hours, and the growth was determined by optical difference with control (without treatment). Amphotericin B (AmB) was used as a gold standard antifungal control. Data representative of one experiment. The bars represent mean with the standard error of the mean (SEM). All the significances are relative to the control group. ^*^ *p* < 0.05; ^**^ *p* < 0.01; ^***^ *p* < 0.005.

First, we assessed a putative antifungal activity of hemolymph against *C. neoformans*. A dose-dependent growth inhibition was observed when the MPH was used (Figure 1A), when compared to untreated control (CTRL). At the lowest dilutions (1:20 and 1:40), fungal growth was inhibited by 75% and 66.6%, respectively. At 1:80 dilution, the inhibition was 33.3%. However, fungal growth seemed unaffected when the higher dilutions were used (1:160 and 1:320). When the RPH was used, similar results were found (Figure 1B). The lowest dilutions (1:20 and 1:40) inhibited fungal growth by 75%. At 1:80 dilution, fungal growth was inhibited by 58.4%, and at 1:160, 16.7%. At 1:320 dilution, no inhibition was observed. 

### Cytotoxicity of hemolymph on RAW264.7 macrophages

**Figure 2.**
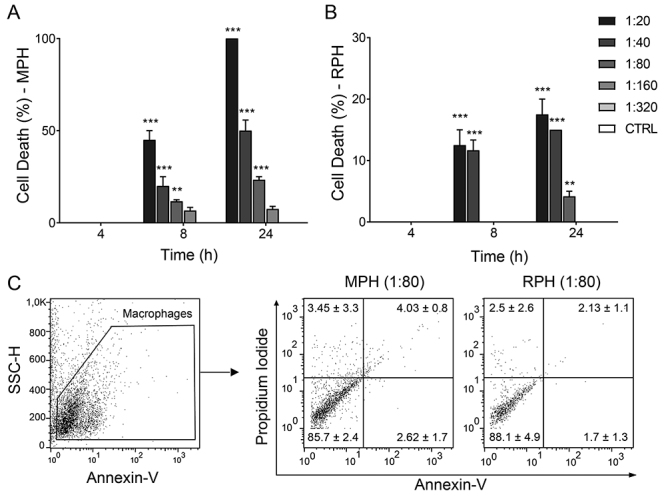
Cytotoxicity test of hemolymph of *Meccus pallidipennis* and *Rhodnius prolixus* on RAW264.7 cells. Cell death rate overtime in the presence of **(A)** *M. pallidipennis* hemolymph (MPH) or **(B)** *R. prolixus* hemolymph (RPH). **(C)** Annexin-V/Propidium Iodide assay of macrophages incubated with MPH or RPH for 4 hours, in the 1:80 dilution. Data representative of one experiment. The bars represent mean with the standard error of the mean (SEM). The significances are relative to the control group. ^*^ *p* < 0.05; ^**^ *p* < 0.01; ^***^ *p* < 0.005.

Next, we aimed to elucidate the cytotoxicity of hemolymph on macrophages. First, a putative toxic effect of hemolymph towards macrophages was assessed (Figure 2). After 4 hours of incubation, no toxicity was observed when macrophages were treated with MPH. After 8h, the MPH killed around 45% of the cells at 1:20, 20% at 1:40 dilution, 11.6% at 1:80 dilution, and 6.6% at 1:160 dilution; the 1:320 dilution seemed not to induce cell toxicity. At 24 hours, 100% of the cells were dead at the lowest dilution (1:20), 50% at 1:40, 23.3% at 1:80 and 7.5% at 1:160 dilution. No toxicity was observed at 1:320 dilution (Figure 2A). No cellular toxicity was detected with RPH 4 hours after the beginning of incubation. After 8 hours, RPH killed up to 12.5% of cells at 1:20 dilution and 11.6% at 1:40 dilution. No toxicity was observed at 1:80, 1:160 and 1:320, respectively. After 24 hours, the RPH killed 17.5% of the cells at 1:20 dilution, 15% at 1:40, and 4.1% at 1:80, with no toxicity detected when the other dilutions were used (Figure 2B). These observations were supported by the Annexin-V/Propidium Iodide assay (Figure 2C), which showed low binding in cells stimulated with both hemolymph samples after 4 hours of incubation , especially at 1/80 dilution. Based on these results, the 1:80 dilution was used in the following experiments for hemolymph of both insects.

### Effects of MPH and RPH on the antigen presentation, expression of co-stimulatory molecules and cytokine production on macrophages

**Figure 3.**
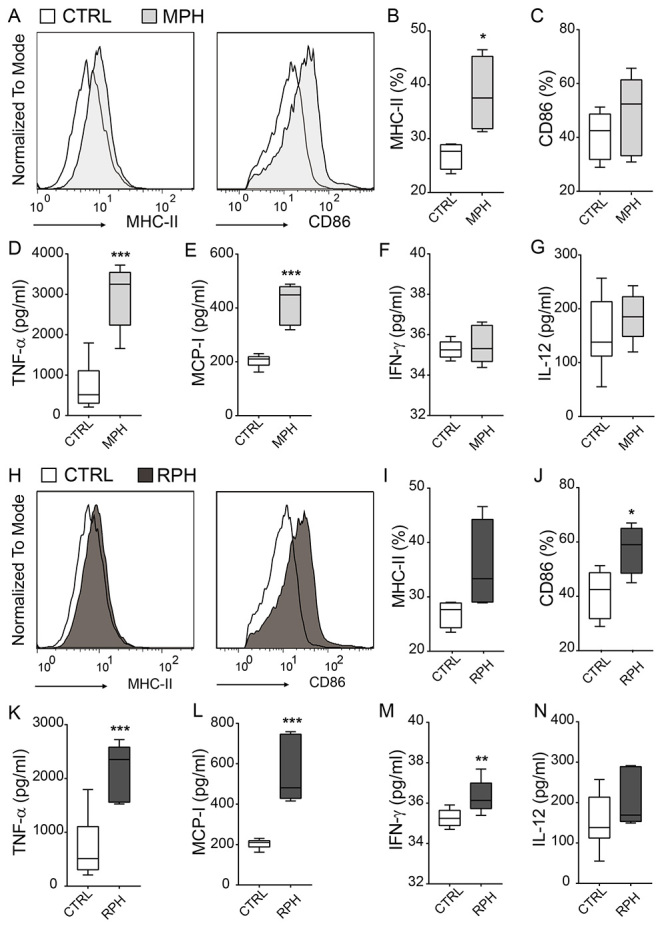
Hemolymph of *Meccus pallidipennis* and *Rhodnius prolixus* modulates the expression of co-stimulatory molecules and cytokine production in RAW264.7 macrophages. **(A-C)** MHC-II and CD86 expression in macrophages stimulated with *Meccus pallidipennis* hemolymph (MPH). **(D)** TNF-α, **(E)** MCP-I, **(F)** IFN-γ, and **(G)** IL-12 production on the supernatant of RAW 264.7 cells stimulated with MPH. **(H-J)** MHC-II and CD86 expression in macrophages stimulated with *Rhodnius prolixus* hemolymph (RPH). **(K)** TNF-α, **(L)** MCP-I, **(M)** IFN-γ, and **(N)** IL-12 were evaluated on the supernatant of RAW 264.7 cells stimulated with RPH. Results representative of two independent experiments. ^*^ *p* < 0.05; ^**^ *p* < 0.01; ^***^ *p* < 0.005.

To address the immunomodulatory activity of MPH and RPH, the expression of MHC-II, CD86, and the production of cytokines were assessed (Figure 3). Treatment with MPH increased the expression of MHC-II, but did not significantly enhance the expression of CD86 in macrophages (Figure 3A, 3B, 3C). In addition, treatment with the MPH increased the production of the pro-inflammatory cytokines TNF-α and MCP-I (Figure 3D, 3E). However, this treatment did not affect the production of IFN-γ and IL-12 (Figure 3F, 3G). Treatment with the RPH increased the expression of CD86 when compared to untreated macrophages; however, it seemed not to affect the expression of MHC-II (Figure 3H, 3I, 3J). Accordingly, the treatment also induced the production of TNF-α, MCP-I, and IFN-γ (Figure 3K, 3L, 3M), with no influence over the production of IL-12 (Figure 3N). These results demonstrate that the hemolymph of both triatomines differently modulate the expression of immunomodulatory molecules in phagocytic cells.

### Hemolymph of hematophagous insects can improve phagocytic and fungicidal activity against *C. neoformans*

**Figure 4.**
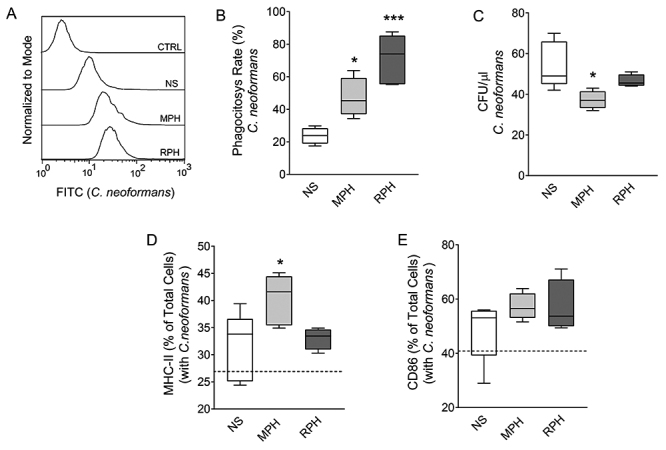
Hemolymph increases the phagocytosis rate and the microbicidal activity in RAW264.7 macrophages. **(A)** Histograms of the internalized fungus (stained with FITC) in RAW 264.7 cells. **(B)** Phagocytosis rate of *C. neoformans* by RAW 264.7 cells. The fungi were incubated with the cells for 4 hours. **(C)** Colony Forming Units (CFU) for μL of lysate from macrophages incubated with *C. neoformans*. RAW 264.7 macrophages were incubated with *C. neoformans* for 48 hours, in the presence or not of both hemolymph samples. **(D-E)** Expression of MHC-II and CD86 in macrophages stimulated with both hemolymph samples. The dashed lines represent the relative expression of the control without *C. neoformans*. Results representative of one experiment. The significances are relative to the NS group. ^*^ *p* < 0.05; ^**^ *p* < 0.01; ^***^ *p* < 0.005.

We aimed to evaluate if the aforementioned immunomodulatory properties of hemolymph of both insects was able to improve the phagocytic and fungicidal activity of macrophages challenged with *C. neoformans*. Regardless the hemolymph used, an enhancement in the phagocytic activity of the macrophages was observed in comparison to non-stimulated cells (NS) (Figure 4A, 4B). Treatment with MPH increased the phagocytic activity by 2-fold, and the RPH increased the phagocytic activity by 3-fold, compared to the NS group (Figure 4B). Also, the effect of hemolymph in improving the killing capacity of macrophages was assessed (Figure 4C). Unstimulated macrophages presented a mean of 54.2 colony-forming units (CFU)/μL of lysate. The MPH impaired the *C. neoformans* colony growth by 31%, compared to the NS (Figure 4C). In turn, there was no significant difference between treatment with RPH and NS cells. Macrophages treated with the RPH presented a mean of 49.75 CFU/μL of lysate. In addition, we evaluated if hemolymph could modulate the MHC-II and CD86 expression in the presence of the fungus. We observed that only the MPH could significantly increase the MHC-II expression in infected macrophages (Figure 4D). Regardless of the hemolymph used, no effects were detected for the expression of CD86 on macrophages during fungal infection compared to untreated cells (Figure 4E). 

### The stimulation of macrophages with MPH or RPH induces the production of MCP-I/TNF-α during *C. neoformans* infection

**Figure 5.**
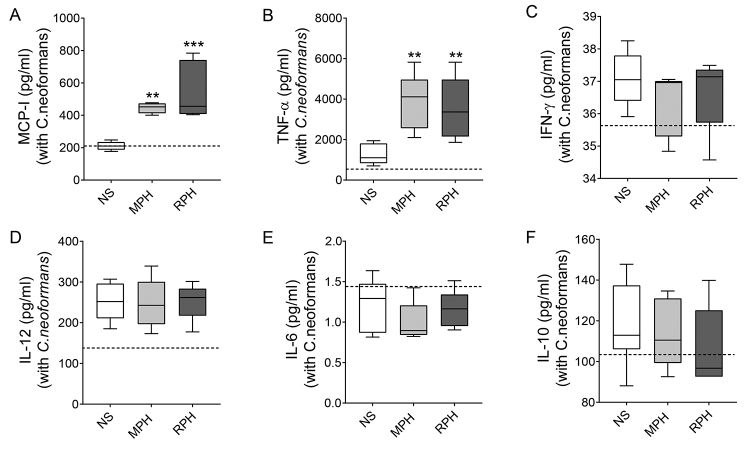
Hemolymph stimuli increase the production of MCP-I/TNF-α in macrophages after 4 hours of incubation. The supernatant of RAW 264.7 cells incubated with *C. neoformans* was collected after 4 hours of culture, and the production of the following cytokines was analyzed: **(A)** MCP-I; **(B)** TNF-α; **(C)** IFN-γ; **(D)** IL-12; **(E)** IL-6 and **(F)** IL-10. Data representative of one experiment. The significances are relative to the NS group. ^*^ *p* < 0.05; ^**^ *p* < 0.01; ^***^ *p* < 0.005.

**Figure 6.**
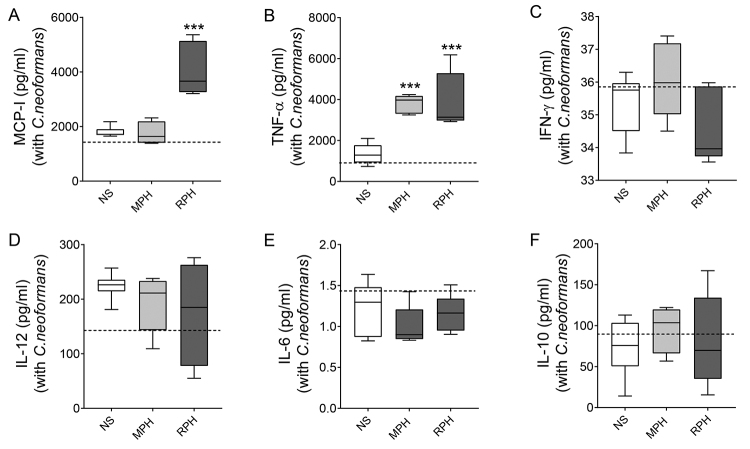
MCP-I/TNF-α levels are still increased 48 hours after incubation with hemolymph. The supernatant of RAW 264.7 cells incubated with *C. neoformans* was collected 48 hours after culture, and the production of the following cytokines was analyzed: **(A)** MCP-I; **(B)** TNF-α; **(C)** IFN-γ; **(D)** IL-12; **(E)** IL-6 and **(F)** IL-10. Data representative of one experiment. The significances are relative to the NS group. ^*^ *p* < 0.05; ^**^ *p* < 0.01; ^***^ *p* < 0.005.

To assess macrophage activation, the production of cytokines in culture supernatant was measured at 4 and 48 hours after the beginning of cultivation with *C. neoformans*. After 4 hours, treatment with both hemolymph samples increased the production of MCP-I and TNF-α when compared to NS (Figure 5A, 5B). However, hemolymph did not influence the production of IFN-γ (Figure 5C), IL-12 (Figure 5D), IL-6 (Figure 5E), and IL-10 (Figure 5F) during infection. To assess the duration of these effects, the production of cytokines was assessed 48 hours after the beginning of cultivation. Again, the production of MCP-I was increased. However, this effect was only observed in the presence of RPH, but not MPH (Figure 6A), when compared to NS cells. Likewise, after 48 hours, treatment with MPH or RPH increased the production of TNF-α when compared to NS (Figure 6B). On the other hand, regardless the hemolymph used, no effects were observed towards the modulation of the following cytokines, IFN-γ (Figure 6C), IL-12 (Figure 6D), IL-6 (Figure 6E), and IL-10 (Figure 6F), in any condition.

## DISCUSSION

For many years, insects' molecules have been studied regarding their potential application in biotechnology, agriculture, and human health. To the best of our knowledge, this is the first work to unveil the presence of molecules in the hemolymph of two different triatomines, *M. pallidipennis* and *R. prolixus*, with immune-stimulatory and antifungal activities against *in vitro* infection of *C. neoformans*. Our results demonstrate a concealed antifungal activity of MPH and RPH in the growth of *C. neoformans*, besides enhanced phagocytic activity of macrophages. We have shown that MPH and RPH could inhibit yeast viability by 70%, in the lower dilution tested. We further demonstrated that MPH and RPH were capable of increasing the phagocytosis of heat-killed yeasts, and the killing activity of viable yeasts by macrophages, especially mediated by the increase of the pro-inflammatory cytokines MCP-I and TNF-α, even after 48 hours of fungal exposure.

The ability of insects to produce microbicidal molecules, especially AMPs, is well known, and the antimicrobial potential of such molecules has been explored for decades [33,34]. Many of these works have studied the antimicrobial activity of hemolymph regarding their bactericidal activity [34-39]. In this context, one of the most prominent therapeutic candidates is cecropin B, an AMP isolated from the hemolymph of the moth larvae *Galleria mellonella*, with *in vivo* and *in vitro* activities against *Pseudomonas aeruginosa* [40]. Regarding triatomines, one of the best-studied species concerning the production of microbicidal molecules is *Triatoma infestans*. This triatomine can produce proteins from the tachykinin family in its hemolymph presenting bactericidal activity against *Micrococcus luteus*, *P. aeruginosa* and *Escherichia coli* [41]. It has been already described that fibrinopeptide A obtained from the *T. infestans* hemolymph, exhibited antimicrobial activity against fungi, including *C. neoformans* [42]; beforehand, antifungal compounds have been isolated from other species as the fly *Lucilia sericata*, and the *G. mellonella* moth larvae [43,44]. 

Once AMPs are the best-described molecules regarding their activity on the immune system and with microbicidal activity in different insects’ families, we can assume they are responsible for the effects observed in this work when MPH and RPH were used. The *C. neoformans* biology has some unique features that can make its treatment difficult. The plasma membrane, the cell wall around it, and the outermost polysaccharide capsule are three outer structures playing a key role in fungus survival, which makes the fungal recognition by the immune system very challenging [19,45]. So far, the fungus has developed several mechanisms of drug resistance, contributing to the low effectiveness of available therapies [46]. The costs for the treatment of cryptococcal meningitis are high. In Uganda, for example, a place with a high incidence of cryptococcal meningitis, it is estimated a cost of US$5-6 million/year to treat such a condition using the gold standard drugs amphotericin B and fluconazole [47]. Once there is still no vaccine or prophylactic therapy for the prevention of *C. neoformans* infection, different approaches are under development or have been proposed, to enhance antifungal responses [48,49]. In this regard, molecules of hematophagous arthropods that could act by distinct mechanisms, directly on the fungi and as an immune stimulator, represents a promising therapeutic approach [50,51]. The results with MPH and RPH are encouraging once it indicates the existence of immune modulatory molecules, influencing the activity of macrophages, and suggests them as alternatives to treat *C. neoformans* infection. However, the mechanisms of action of the majority of AMPs on *C. neoformans* and other fungi have not been fully elucidated. Indeed, some of the known mechanisms in this scenario include disruption of microbial cellular membranes, metabolic disturbances, and ROS-mediated apoptosis [52-54]. Despite the direct effects of hemolymph towards fungal growth, the molecule(s) accounting for these effects was/were not identified in the present study.

Aside their activity against microorganisms, AMPs can also be cytotoxic to mammalian cells. Previously data have demonstrated that the cytotoxicity of cationic AMPs in PBMCs is attributed to the induction of apoptosis [55]. In addition, invertebrates, can produce some other molecules with cytotoxic activity including reactive intermediates of oxygen and nitrogen, lectins, cytokine- and complement-like molecules, and quinoid intermediates of melanin [56]. Thus, assessing cell toxicity was of paramount importance to elucidate if RPH and MPH can present any toxic effects to macrophages. Indeed, our results showed that both hemolymph samples presented only low toxicity to macrophages, while acting directly on the pathogen *in vitro*. Still, the *in vivo* evaluation of the compounds would be valuable to assure these results in experimental models of fungal infection, before performing studies on human subjects.

We also demonstrated the effects of hemolymph on the modulation of MHC-II and CD86 on macrophages treated with MPH and RPH. These molecules play a crucial role in antigen presentation, thus linking innate to adaptive immune response. MHC-II is crucial to antigen presentation, while CD86 works as a co-stimulatory molecule, binding to CD28 on T lymphocytes [57]. Different microorganisms have developed strategies to evade the host immune response, which includes *C. neoformans.* Among these mechanisms, it is possible to highlight the negative modulation of MHC-II in different subsets of pulmonary phagocytes during *C. neoformans* infection [58]. Similarly, encapsulated yeasts reduced the expression of MHC-II and CD86 in infected macrophages [59-61]. The diminished expression and activity of these key molecules in antigen presentation results in poor inflammatory and T cell activation [61]. In our study, MPH and RPH induced the expression of MHC-II and CD86, respectively, in macrophages. The higher expression of such molecules reinforces the immunomodulatory activity of hemolymph besides suggesting the existence of compounds able to constrain the mechanisms of immune evasion used by the fungus to suppress host’s immune response. 

Another prominent effect observed during the treatment with hemolymph was on cytokine production. The hemolymphs of both insects increased the production of MCP-I and TNF-α on macrophages, even after 48 hours of stimulation. MCP-I and TNF-α are seminal cytokines produced at the initial phase of the inflammatory response [20]. During *C. neoformans* infection, the production of the chemokine MCP-I (also known as CCL2) by alveolar macrophages exerts a key role in chemotaxis, stimulating the migration of mononuclear cells from the bloodstream to the infection site [62-64]. Thus, an increase in MCP-I, such as those observed when RPH and MPH were used in the present work, indicates a higher mobilization of innate and adaptive cells to the infection sites, which may contribute to a better fungal control and infection outcome. TNF-α is one of the major molecules produced by the early innate immune cells during the inflammatory response [65]. The secretion of TNF-α, along with some other molecules (including but not exclusively GM-CSF), is an important mediator of resistance against encapsulated pathogens, including *C. neoformans*, by stimulating phagocytosis and pathogen killing [66]. In addition, TNF-α is an important mediator of the adaptive response against the fungus. The production of TNF-α during the immune response against *C. neoformans* infection is critical for the development of protective CD4 T cell immunity in the lungs and extrapulmonary sites [26,48,49,67-70]. As MPH and RPH induced the modulation of two key pro-inflammatory molecules in the response against *C. neoformans*, with known activity towards innate and adaptive immunity, it is feasible to assume they are also able to improve the immune response, and perhaps infection outcome, against the fungus *in vivo*, reducing disease burden and related-costs. However, to test this hypothesis, further studies addressing the *in vivo* impact of such approach must be conducted.

This is the first time that molecules from MPH and RPH were shown to constrain *C. neoformans* growth, concomitantly increasing macrophages' activity and function. So far, a lot has been studied about arthropods-derived molecules regarding their use for the human purpose [71,72]. Alloferons, which are peptides isolated from the blow fly *Calliphora vicina*, have presented *in vivo* antiviral and antitumoral activities, as well as *in vitro* stimulatory activities on natural killer lymphocytes [73]. This peptide has been tested in clinical trials for treating viral infection in humans, and have generated the world’s first medicinal synthetic product based on a natural occurring molecule from arthropods [74]. A previous study from our group shows that saliva from different species of triatomines is capable of inhibiting the differentiation and maturation of LPS-stimulated dendritic cells [29]. Also, some authors have recently described a protease activity from the saliva of *Triatoma infestans* acting as a vasodilator, which can stimulate inflammatory activity [75]. In this present work, we have demonstrated that MPH and RPH have molecules with immunomodulatory activity, capable of improving the production of pro-inflammatory cytokines and the function of macrophages. Despite our promising results concerning the antimicrobial activity and immune modulatory properties of the hemolymphs, this study has some limitations: the lack of elucidation towards a putative effect of hemolymph on macrophage polarization; the biochemical and molecular characterization of the molecule(s) accounting for the effects observed; and the lack of in vivo data to fully address the potential of hemolymph to treat *C. neoformans* infection. 

## CONCLUSION

MPH and RPH can act directly on *C. neoformans*, besides stimulating macrophages to increase the production of pro-inflammatory cytokines and expression of co-stimulatory molecules, resulting in increased phagocytosis and fungal death. The ambiguous role of hemolymph, constraining microbial growth besides stimulating key components in immune response, suggests the existence of molecules to be explored in the future as therapeutic approaches to treat *C. neoformans* infection. Considering the lack of further studies describing the immunomodulatory activity of hemolymph, more experiments are required to elucidate the molecules behind these effects and their activity on other immune cells. 

### Abbreviations

AmB: amphotericin B; AMP: antimicrobial peptide; CN: *Cryptococcus neoformans*; MPH: *Meccus pallidipennis* hemolymph; RPH: *Rhodnius prolixus* hemolym

## Supplementary material

The following online material is available for this article:

Additional file 1. Gate strategies and plots for the MHC-II/CD86 expression in RAW264.7 cells. **(A)** Strategy for macrophage identification and **(B-C)** histograms of MHC-II and CD86 expression.

Additional file 2. Gate strategies and plots for the *C. neoformans* phagocytosis in RAW264.7 cells. **(A)** Strategy for FITC^+^ macrophages identification and **(B)** dot plots of FITC^+^ macrophages in the different groups.
