# Supplementary material for: Hemolymph of triatomines presents fungistatic activity against Cryptococcus neoformans and improves macrophage function through MCP-I/TNF-α increase
Source: J Venom Anim Toxins Incl Trop Dis. 2022 Jul 18;28:e20210124. doi: 10.1590/1678-9199-JVATITD-2021-0124 (PMC9302513; doi:10.1590/1678-9199-JVATITD-2021-0124)
Supplement: Additional file 2. [file 1678-9199-jvatitd-28-e20210124-s2.pdf]

J Venom Anim Toxins incl Trop Dis.

https://doi.org/10.1590/1678-9199-JVATITD-2021-0124

Supplementary Material to "Hemolymph of triatomines presents fungistatic activity against *Cryptococcus neoformans* and improves macrophage function through MCP-I/TNF-α increase"

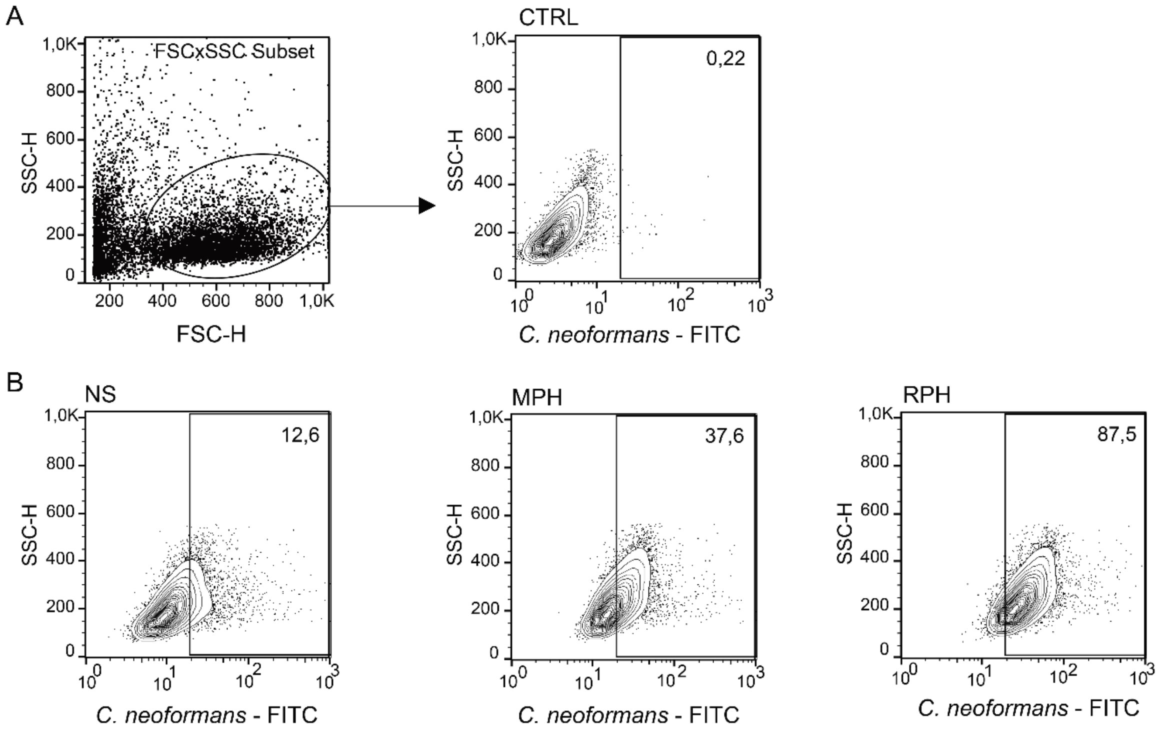

Additional file 2. Gate strategies and plots for the C. neoformans phagocytosis in RAW264.7 cells. (**A**) Strategy for FITC+ macrophages identification and (**B**) dot plots of FITC+ macrophages in the different groups.
